# Updates in heart failure: what last year brought to us

**DOI:** 10.1002/ehf2.12385

**Published:** 2018-12-20

**Authors:** Elisabetta Dinatolo, Edoardo Sciatti, Markus S. Anker, Carlo Lombardi, Nicolò Dasseni, Marco Metra

**Affiliations:** ^1^ Department of Medical and Surgical Specialties, Radiological Sciences and Public Health University of Brescia Brescia Italy; ^2^ Division of Cardiology and Metabolism, Department of Cardiology, Berlin‐Brandenburg Center for Regenerative Therapies (BCRT), DZHK (German Centre for Cardiovascular Research), partner site Berlin Charité—Universitätsmedizin Berlin Berlin Germany

## Epidemiology

Heart failure (HF) is common, and HF with preserved ejection fraction (HFpEF) has become its most frequent clinical presentation because of ageing of the population and decreasing prevalence of coronary artery disease (CAD).^1^, ^2^ An analysis of all studies using echocardiography to estimate the prevalence of cardiac dysfunction in subjects aged ≥60 years showed a median prevalence of 36.0% (range 15.8–52.8%) and 5.5% (range 3.3–9.2%) for ‘isolated’ left ventricular (LV) diastolic dysfunction and LV systolic dysfunction, respectively, and a median prevalence of 4.9% (range 3.8–7.4%) and 3.3% (range 2.4–5.8%) for symptomatic HFpEF and HF with reduced ejection fraction (HFrEF).^3^

### Outcomes

Outcomes of patients with HF remain poor. The European Society of Cardiology (ESC) HF Long‐Term Registry collected data of 12 440 patients with HF, 59.5% outpatients and 40.5% hospitalized patients for acute HF (AHF), enrolled from 211 cardiology centres in 21 European and/or Mediterranean countries.^4^ The 1 year all‐cause mortality rate was 6.4% for ambulatory patients and raised to 23.6% for those hospitalized for AHF. The combined endpoint of 1 year mortality or HF hospitalization occurred in 14.5% of outpatients and 36% of hospitalized patients. A primary care‐based cohort study in Scotland compared the 5 year survival of patients with HF with that of the most common causes of cancer. The 5 year survival of patients with HF was of 55.8% in men and 49.5% in women, and it was better than that of patients with lung cancer, colorectal cancer, and, in women, ovarian cancer but worse than that of male patients with prostate cancer and of female patients with breast cancer.^5^

### Temporal trends

Temporal trends in hospitalization rates and outcomes of patients with HF were examined in multiple studies based on national databases from European countries. In general, all studies confirm better survival of patients with a new diagnosis of HF. Hospitalization rates have more variable trends because of the opposing influence of the increase in the absolute number of HF patients and their better outcome.

The most optimistic data came from Denmark. In this country, the standardized annual rate of HF hospitalizations decreased by an average of 3.5% each year, starting from the year 2000, and the 1 and 5 year mortality rates declined from 45 to 33% and from 59 to 43%, respectively, in the years 2008–2012, compared with the years 1983–1987.^6^ In contrast, in France, despite a decrease in mortality rates by 3.3% each year, from 2000 to 2010, HF hospitalization rates remained stable. Sex differences were also noted with a lower decrease in HF hospitalizations and a larger decrease in mortality rates in men vs. women.^7^

Data from Germany and from Slovenia show an increase in HF hospitalizations, mostly related with ageing of the general population. In Germany, the absolute number of HF‐related hospitalizations increased by 65.4%, 28.4% after age standardization, in the year 2013, compared with 2000. Accordingly, the absolute number of HF‐related hospital days increased by 22.1%, despite a 25.9% decrease in the average length of stay from 14.3 to 10.6 days. In‐hospital mortality rates also remained high (9.3% in 2013). These trends of increased HF hospitalizations and high in‐hospital mortality rates affected mostly the patients aged >65 years.^8^ The role of age is shown also by the data collected in Slovenia where age‐standardized HF hospitalization rates decreased by 7.1%, whereas the absolute number of HF hospitalizations increased by 19.8% between the years 2004 and 2012.^9^

### Geographical distribution

Heart failure has a worldwide clinical impact. Geographical differences are therefore more and more important.

Southeast Asia is home of a growing population of >600 million people with a relatively younger age, compared with Western countries. Subjects in Southeast Asia have a high prevalence of risk factors, particularly hypertension, smoking, physical inactivity, and diabetes, although a lower prevalence of overweight/obesity. Epidemiological trends in Singapore showed a sharp 38% increase in age‐adjusted HF hospitalizations. Compared with patients in Western countries, these patients are younger and have high mortality rates, and HF treatment is still largely underused.^10^

Japan has the oldest population in the world with >25% of the population aged ≥65 years, and this has an impact on the growing prevalence of HF.^11^ The estimated prevalence of HF is ~1 million people in Japan and >4 million in China. Mortality rates after discharge from an HF hospitalization are lower in Japan than in Western countries with 1 year rates of 9–12%. The very long length of stay (15–21 days) of an HF hospitalization in Japan likely contributes to this finding.^11^, ^12^, ^13^

## Phenotypes and diagnostic assessment

The new ESC HF guidelines have established new criteria for the diagnosis of HFpEF and HF with mid‐range ejection fraction (HFmrEF). They are based on the presence of symptoms and/or signs of HF, an LV ejection fraction (EF) of 40–49% for HFmrEF and ≥50% for HFpEF, and signs of cardiac dysfunction, including high brain natriuretic peptide (BNP) or N‐terminal pro‐BNP (NT‐proBNP) plasma levels and signs of structural heart disease, LV hypertrophy, or left atrial dilatation, and/or of LV diastolic dysfunction, namely, an abnormal E/e′ ratio. These criteria remain, however, less simple than for HFrEF, and the diagnosis can be further complicated by the prominent role of co‐morbidities. A diagnostic algorithm based on clinical and echo‐Doppler criteria has recently been proposed.^14^

In symptomatic patients with normal LVEF and normal pulmonary artery wedge pressure (PAWP) at rest, right heart catheterization during exercise can unmask diastolic dysfunction showing an increase in PAWP during exercise.^15^ It is still debated whether an echocardiographic assessment can substitute invasive haemodynamic measurements during exercise.^16^, ^17^ Using resting and exercise haemodynamic data and cardiac imaging, Obokata *et al*.^18^ have described a distinct obese phenotype of HFpEF, characterized by worse exercise capacity, higher biventricular filling pressures during exercise, and reduced pulmonary artery vasodilator reserve.

Abnormalities of LV systolic function can be found despite a normal EF. A reduced LV global longitudinal strain (GLS) with an increased LV circumferential strain was shown in HFpEF and was associated with higher PAWP.^19^ Measurement of LV longitudinal strain may be particularly useful in specific patients' groups at high risk for HF, such as those with valve disease or those undergoing chemotherapy.^20^, ^21^

### Heart failure with mid‐range ejection fraction

The recent ESC HF guidelines have introduced the new category of HFmrEF. The authors wrote ‘we believe that identifying HFmrEF as a separate group will stimulate research into the underlying characteristics, pathophysiology and treatment of this population’.^2^ Such a prophecy was fulfilled. The clinical characteristics and biomarker profiles of the patients with HFmrEF are, in general, intermediate between those of the patients with HFrEF and HFpEF. However, the prevalence of CAD is generally similar to that of patients with HFrEF.^22^, ^23^, ^24^

Outcomes of patients with HFmrEF are generally better than in patients with HFrEF and similar to that of HFpEF patients.^23^, ^25^ In contrast, the response to treatment seems similar to that of patients with HFrEF. In TOPCAT (Aldosterone Antagonist Therapy for Adults With HFpEF) trial, spironolactone reduced outcomes in patients with lower baseline LVEF (<50%).^26^ Similarly, in Candesartan Cilexetil in HF Assessment of Reduction in Mortality and Morbidity (CHARM), candesartan reduced the primary outcome also in patients with HFmrEF with an effect on recurrent HF hospitalizations that remained significant until EF was ≤60%.^27^ Beta‐blockers improved LVEF and reduced untoward outcomes only in patients with reduced LVEF in an individual patient meta‐analysis of previous controlled trials.^28^ Digoxin reduced HF hospitalizations and the composite endpoint of deaths and HF hospitalizations in patients with HFrEF, not significantly in those with HFpEF, and at an intermediate level in those with HFmrEF.^29^

Thus, it seems that HFmrEF is a ‘milder form’ of HFrEF including patients, mainly with CAD, who had only mild myocardial injury or who have recovered from more severe myocardial dysfunction. Consistently, these patients also have a better prognosis than those with HFrEF and, as the mechanisms of dysfunction are the same, respond to the same drugs active in patients with HFrEF. However, perhaps the greatest advantage of the introduction of the HFmrEF category is to highlight the different characteristics of patients with an LVEF ≥ 50%, a view shared also in the more recent Australian and New Zealand HF guidelines that use a cut‐off of 50% to differentiate between HFrEF and HFpEF.^30^

## Clinical assessment

### Heart failure prediction in asymptomatic subjects

Efforts have been dedicated to the identification of asymptomatic subjects at higher risk of HF development. Earlier initiation of medical therapy and better control of risk factors may prevent HF and, ultimately, improve survival in these patients.^1^, ^2^ Both echocardiographic parameters and biomarkers have been evaluated.

In a large cohort of asymptomatic subjects ≥65 years old, with ≥1 HF risk factor, who underwent echocardiographic screening, Yang *et al*.^31^ found a prevalence of 13% for LV hypertrophy, 12% for an abnormal E/e′, 33% for an impaired GLS, and 31% for left atrial enlargement, and these parameters were independent predictors of new HF. Left ventricular mass and GLS, but not the other parameters, significantly reclassified individuals compared with traditional risk factors.

Strategies based on NT‐proBNP measurements were proven as effective for the prevention of HF development. Abouezzeddine *et al*.^32^ compared the predictive value for HF and major cardiac events of multiple biomarkers in a community cohort in Olmsted County. Only NT‐proBNP and high sensitivity (hs) cardiac troponin T (cTnT) had an independent predictive value, compared with clinical variables. A strategy based on screening with NT‐proBNP assays in the general population was cost‐effective and compared favourably with established interventions.^32^

### Symptoms and signs in chronic heart failure

Body weight remains an important prognostic variable. According to the obesity paradox, increased body weight is associated with better outcomes. Body surface area might be preferred to body weight, and in the ESC HF Long‐Term Registry, body surface area had an inverse relation with total and cardiovascular mortality but not with hospitalizations.^33^ Patients with morbid obesity do not have the same survival advantage of their obese or overweight counterparts.^34^ In the AHF Global Survey of Standard Treatment registry, body weight had a U‐shaped relationship with mortality with the lowest values in overweight subjects. However, this relationship vanished after adjustment for covariates. Changes in body weight are also used to monitor fluid status in patients with decompensated HF. However, their sensitivity is lower compared with echocardiographic parameters and NT‐proBNP plasma levels.^35^, ^36^

Cachexia, sarcopenia, and unintentional weight loss are associated with poorer outcomes.^37^ In addition, frailty, assessed as low physical activity, weight loss, slow walking speed, weak grip strength, and exhaustion, was an independent predictor of early disability, long‐term mortality, and re‐hospitalizations.^38^, ^39^

Blood pressure is another parameter with a paradoxical inverse relationship with outcomes in HF. In addition to absolute values, the changes in blood pressure may also have prognostic significance. A long‐term reduction in systolic blood pressure greater than ±10 mmHg/year was associated with an increased risk of death or heart transplantation (1.8 and 2.0, respectively).^40^ Pulse pressure is related with vascular compliance as well as with cardiac output. In a cohort study of consecutive HFpEF patients, pulse pressure was significantly and positively correlated with pulse wave velocity and LV stroke volume index, and patients in the lowest (<45 mmHg) and the highest (>75 mmHg) pulse pressure quintiles had a significantly higher risk of cardiovascular and HF‐related events.^41^

Heart rate is an independent predictor of mortality in HF. A resting heart rate >70–75 b.p.m. has been identified as a major risk factor for poorer outcomes in patients with HFrEF, AHF, and many other conditions, including cancer.^42^, ^43^, ^44^ Selective heart rate lowering in patients with HF is now indicated in the guidelines both from ESC, the USA, Australia, and New Zealand.^2^, ^30^, ^45^

### Acute heart failure

Acute HF is associated with a dramatic increase in the risk of subsequent death or re‐hospitalization.^2^ Many studies are focused on the prognostic stratification of these patients. Precipitating factors are important. In an observational study, precipitating factors were classified in four main groups: acute coronary syndrome, atrial fibrillation, acute pulmonary disease, and other causes. Atrial fibrillation and acute coronary syndrome were associated with more readmissions. Acute coronary syndrome and pulmonary disease were associated with higher mortality in the short term (1 week) and medium term (3 weeks), respectively.^46^, ^47^

Data from the ESC HF Long‐Term Registry suggest that the clinical phenotype at admission can be used to stratify patients and predict 1 year mortality, being this higher in cardiogenic shock, right HF, pulmonary oedema, and decompensated HF than with acute coronary syndromes and hypertensive HF.^48^ Interestingly, patients who survived at least 6 months post‐discharge represented a more homogeneous group, and their 1 year outcome was less influenced by their initial clinical profile or systolic blood pressure at admission.^48^

Worsening HF, an event occurring in 7–30% of patients during hospitalization, is associated with increased re‐hospitalization and post‐discharge mortality rates and has been used as an endpoint in clinical trials.^12^, ^49^, ^50^ Also, an increased length of stay may predict subsequent worse outcomes, although geographical differences may reduce its potential value as an endpoint for clinical trials.^50^

### Risk scores

BIOSTAT‐CHF (A systems BIOlogy Study to TAilored Treatment in Chronic HF) was a large, multicentre, prospective, observational study including 2516 patients from 11 European countries with worsening HF symptoms and 1738 patients from Scotland as validation cohort.^51^ Based on the variables collected, a risk score for outcomes was developed. The five strongest predictors of mortality were older age, higher blood urea nitrogen, high NT‐proBNP, lower haemoglobin, and failure to prescribe a beta‐blocker. The five strongest predictors of HF hospitalization were older age, previous HF hospitalization, peripheral oedema, lower systolic blood pressure, and lower estimated glomerular filtration rate.^52^

## Cardiac imaging and invasive haemodynamics

Imaging methods have been focused mainly on pulmonary hypertension, right ventricular (RV) function, and mitral regurgitation. These measurements are useful in both HFrEF and HFpEF.

Pulmonary hypertension remains a major independent determinant of outcomes.^53^ Detection of increased pulmonary artery pressure (PAP) through wireless ambulatory monitoring is among the few procedures that has significantly reduced HF hospitalizations.^54^, ^55^ The 2015 ESC/ERS guidelines differentiate two different haemodynamic subsets of pulmonary hypertension due to left heart disease, based on levels of pulmonary vascular resistance and diastolic pressure gradient: isolated post‐capillary pulmonary hypertension and combined post‐capillary and pre‐capillary pulmonary hypertension.^56^ The value of the diastolic pressure gradient is, however, controversial as it may become negative because of large V waves in PAWP tracings due to mitral regurgitation, and these patients may have a better outcome than those with combined pre‐capillary and post‐capillary pulmonary hypertension.^57^, ^58^, ^59^

Pulmonary hypertension and RV dysfunction are present in a significant proportion of patients with HFpEF.^60^ Their prevalence is 68% and 18–28%, depending on the measurement used. They were both associated with mortality in a systematic review and meta‐analysis.^61^ Studies based on the assessment of RV function by cardiac magnetic resonance have also shown significant associations with outcomes in specific patient groups such as those with HFpEF.^62^

Echocardiographic parameters are often used as surrogates of LV and pulmonary pressures. However, a poor‐to‐moderate correlation was found among echocardiographic parameters (E/E′, isovolumetric relaxation time, left atrial reservoir strain, and RV wall thickness) and invasive haemodynamic measurement [PAWP, LV end‐diastolic pressure (LVEDP), mean PAP, and PAP], independently from the presence of atrial fibrillation.^16^ Patients with atrial fibrillation had PAWP values higher than LVEDP, whereas PAWP was lower than LVEDP in patients in sinus rhythm.^17^

Right ventricular function, particularly when assessed with indexes that correct for pulmonary pressure, such as the tricuspid annular plane systolic excursion/systolic PAP ratio, the RV longitudinal strain/systolic PAP ratio, or the tricuspid annular plane systolic excursion × transtricuspid systolic gradient product, is independently related with mortality in addition to, or differently from, pulmonary hypertension alone. Its prognostic value is confirmed in both HFrEF and HFpEF, although its determinants differ. Improvement in RV function after treatment is predictive of better outcomes compared with persistent or worsened dysfunction during follow‐up.^63^, ^64^, ^65^

## Biomarkers

Multiple novel biomarkers, assessing different mechanisms of HF, cell death, fibrosis, neurohormonal activation, inflammation, and other organ damage, have been introduced.^66^ To date, only natriuretic peptides are recommended in current ESC guidelines.^2^ They are sensitive markers of increased myocardial wall stress and hence increased LV diastolic pressure and congestion. However, their value is influenced by multiple variables among whom age and heart rhythm have a major role. Their diagnostic accuracy is reduced in elderly subjects.^67^ Atrial fibrillation causes an increase in BNP levels and reduces their sensitivity for the detection of changes induced by treatment.^68^ Lastly, two prospective randomized trials aimed at the evaluation of the clinical impact of serial measurements of BNP levels have failed to show beneficial effects on outcomes, likely because optimization of medical treatment occurred also in the control group.^69^, ^70^

Mid‐regional pro‐adrenomedullin (MR‐proADM) is another marker related with myocardial stress. The Interdisciplinary Network HF programme enrolled 1022 patients hospitalized for acute systolic HF and followed them for 18 months. High MR‐proADM was associated with more impaired LV function, higher co‐morbidity burden, lower doses of HF medications, and lower likelihood of LV reverse remodelling. Compared with natriuretic peptides, MR‐proADM had superior prognostic significance and improved Cox regression models including natriuretic peptides and was the only biomarker predicting also non‐cardiac death. Six month MR‐proADM enhanced models including baseline MR‐proADM (*P* < 0.001) for prediction of all‐cause death [net reclassification index: 0.48, 95% confidence interval (CI) 0.19–0.78]. Serial MR‐proADM measurements after 6 months enhanced risk assessment. Cardiac troponin is a sensitive marker of myocardial injury, and its value for the prognostic assessment of patients with HF is now approaching that of natriuretic peptides. It is an independent predictor of cardiovascular outcomes in subjects at risk of cardiac disease as well as in HF patients either ambulatory or recently hospitalized.^71^, ^72^, ^73^, ^74^ In a recent individual patient data meta‐analysis including data from 10 studies and 9289 patients with chronic HF, hs cTnT was added to a prognostic model including established risk markers (sex, age, ischaemic vs. non‐ischaemic aetiology, LVEF, estimated glomerular filtration rate, and NT‐proBNP) and significantly improved risk prediction for all‐cause and cardiovascular mortality and cardiovascular hospitalizations.^75^

Biomarkers allow the assessment of co‐morbidities, such as kidney dysfunction and iron deficiency. Serum creatinine changes maintain a major role for the prognosis of patients with acute and chronic HF. Excessive diuresis, hypotension, and initiation of renin–angiotensin–aldosterone inhibitors may cause increases in serum creatinine unrelated with prognosis. These conditions must be considered for the interpretation of the serum creatinine changes.^76^, ^77^, ^78^ Cystatin C is useful for the detection of impaired glomerular filtration rate at an earlier stage, before serum creatinine increases.^76^ Markers of renal tubular function were studied for the early detection of tubular damage and kidney injury. In AHF, plasma kidney injury molecule‐1 predicted HF re‐hospitalization, while urinary kidney injury molecule‐1, together with urinary neutrophil gelatinase‐associated lipocalin, predicted the development of true worsening renal function.^79^, ^80^ The ESC HF guidelines mandate the assessment of iron deficiency through measurements of serum ferritin and transferrin saturation as its treatment may improve quality of life and exercise capacity and reduce hospitalizations in patients with HFrEF.^2^, ^81^, ^82^, ^83^

Each biomarker measures different pathways. A multi‐marker strategy, based on new platforms that allow the measurement of up to 48 or 96 different biomarkers, allows the detection of the mechanisms involved in different patients with HF. For instance, network analysis of a panel of 48 different biomarkers in patients with AHF with or without diabetes showed a strong cluster of biomarkers related with inflammation and fibrosis, such as interleukin‐6, periostin, and C‐reactive protein (CRP), suggesting a specific activation of these pathways, in diabetic patients but not in non‐diabetic patients.^84^ An analysis of the prognostic value of 44 different biomarkers in patients with AHF showed an 11% increase in C‐index to 0.84 and 0.78 for 30 and 180 day all‐cause mortality with the combination of blood urea nitrogen, chloride, interleukin‐6, troponin I, soluble suppression of tumorigenicity*‐*2 (sST2) and vascular endothelial growth factor receptor‐1 into a clinical model.^85^ Serial measurements are also important and provide better prognostic assessment compared with baseline values only. A repeat measurement as early as Day 2 was adequate for NT‐proBNP and cystatin C in terms of maximizing discriminatory accuracy, and further measurements on Days 14 and 60 provided added value for hs cTnT, growth differentiation factor‐15, sST2, and hs CRP.^86^

Studies comparing new biomarkers with traditional ones and clinical assessment are still needed. Jackson *et al*.^87^ evaluated the incremental prognostic value of multiple novel biomarkers in 628 patients recently hospitalized with decompensated HF. At multivariable analysis, MR‐proADM, hs cTnT, combined free light chains, hs CRP, and sST2 had additional prognostic value compared with traditional clinical signs and biomarkers.^87^ In another study, neither BNP nor cTnT measured at admission improved outcome prediction, compared with clinical data.^88^ MicroRNAs are another major area of research not only as potential targets of treatment but also as biomarkers. Plasma levels may have prognostic value in acute or chronic HF and may change after treatment.^89^, ^90^

## Co‐morbidities

Co‐morbidities are a major determinant of clinical presentation, outcomes, and treatment of patients with HF.^91^, ^92^ Ageing of the patient population has led to their increased prevalence in the last years.^6^, ^9^ They seem more important in HFpEF and HFmrEF, whereas HFrEF patients have more often CAD as the main cause of HF.^23^, ^24^, ^93^ Co‐morbidities are usually divided into cardiovascular, such as hypertension,^94^ CAD,^95^ atrial fibrillation,^96^ stroke,^97^ and non‐cardiovascular, such as cancer,^98^, ^99^ chronic renal dysfunction,^79^, ^100^ obstructive lung disease,^101^, ^102^, ^103^ sleep apnoea,^104^, ^105^ iron deficiency,^106^, ^107^ anaemia,^108^, ^109^, ^110^ sarcopenia,^111^, ^112^ anorexia,^39^, ^113^ frailty,^38^, ^114^ cachexia,^115^, ^116^ liver dysfunction,^117^diabetes mellitus,^84^, ^118^ obesity,^34^, ^119^, ^120^ and psychiatric disorders.^121^ They are often associated with an increased risk of HF in initially asymptomatic patients as they may cause or favour the development of cardiac dysfunction. Second, they cause more severe symptoms and are associated with an increased rate of major events, including cardiovascular and all‐cause hospitalizations, and death, once they occur in patients with HF. Their effect may be direct or through a negative impact on the administration of evidence‐based treatment, such as is the case of renal dysfunction, which may contraindicate inhibitors of the renin–angiotensin–aldosterone system.^91^ Their extensive assessment goes beyond the aims of this article. They are considered in the paragraphs about diagnosis and treatment.

## Prevention

Control of risk factors for CAD remains the mainstay for prevention of development of HF. Other general measures, such as pneumococcal vaccination, may be important, especially in elderly subject.^122^

The ESC guidelines have included, for the first time, antidiabetic treatment for the prevention of HF. Namely, empagliflozin, a sodium glucose transporter (SGLT‐2) inhibitor, has reduced HF related events and mortality in patients with diabetes at high risk of cardiovascular events, and it is now recommended for HF prevention.^2^, ^123^, ^124^ In contrast, the glucagon‐like peptide‐1 analogue liraglutide, despite its beneficial effects on stroke, myocardial infarction, and mortality in diabetic patients,^125^ did not improve LV systolic function and had no effects on cardiac events or increased them, numerically, in two prospective placebo‐controlled randomized trials in stable chronic HFrEF patients with and without diabetes.^126^, ^127^ Among the dipeptidyl peptidase‐4 inhibitors, saxagliptin was associated with an increase in HF hospitalization, whereas alogliptin and sitagliptin were not. No effect on major cardiovascular outcomes was found with all these agents.^128^, ^129^, ^130^ Insulin administration was associated with worse outcomes in an analysis of 24 012 patients with HF and diabetes from four large randomized trials and of 103 857 patients from an administrative database. Evidence from prospective studies would be needed.^131^

## Medical treatment

No major changes have occurred in current evidence for medical treatment since the results of SHIFT (Systolic HF Treatment with the I(f) Inhibitor Ivabradine Trial) and PARADIGM‐HF (A Multicentre, Randomized, Double‐blind, Parallel Group, Active‐controlled Study to Evaluate the Efficacy and Safety of LCZ696 Compared to Enalapril on Morbidity and Mortality in Patients With Chronic HFrEF).^132^, ^133^ The focus is now on the implementation of evidence‐based treatment and how much the results of clinical trials can be translated into clinical practice.^134^, ^135^

Treatment of HFpEF remains disappointing. It is hypothesized that reduced nitric oxide availability may cause HFpEF so that drugs increasing it may improve these patients.^136^ However, recent controlled trials with sildenafil as well as with organic nitrates have given neutral results.^137^, ^138^, ^139^ Inorganic nitrites have improved arterial compliance, exercise haemodynamics, and exercise capacity in small studies.^140^, ^141^, ^142^ The larger INDIE‐HFpEF (Inorganic Nitrite Delivery to Improve Exercise Capacity in HFpEF) trial has been designed, though with neutral results, as recently presented by Borlaug at the American College of Cardiology (ACC) 2018 Annual Scientific Sessions (unpublished data).^143^ Slowing heart rate is another potential target of HFpEF treatment. However, in EDIFY (prEserveD LVEF chronic HF with ivabradine studY) (EDIFY), slowing heart rate with ivabradine had no effect on parameters of LV diastolic function and NT‐proBNP levels.^144^

Based on the results of Phase II studies, above all with respect to patient‐reported outcomes as endpoints, trials with guanylate cyclase activators are ongoing.^145^, ^146^ Results of a major outcome trial with sacubitril/valsartan are expected soon.^147^ Despite the neutral results with the primary endpoint of TOPCAT, HF hospitalizations were reduced, and geographical differences had a major impact.^148^ There are, thus, both pathophysiological mechanisms and trial results that suggest that mineralocorticoid receptor antagonists (MRA) should have beneficial effects in HFpEF patients. A trial with a pragmatic study design in HFpEF patients enrolled in a Swedish registry is ongoing.^149^

### Acute heart failure

Congestion is the main cause of hospitalization for HF patients and diuretics are the mainstay of treatment.^2^ Diuretic treatment is still based on furosemide administration. Its initial dose and mode of administration, bolus vs. continuous infusion, have not influenced outcomes in a pivotal trial.^150^ Congestion relief may be monitored by different tools, including clinical signs, imaging methods and laboratory exams.^151^ A recent prospective study has shown the value of haemoconcentration as a simple but sensitive tool to detect congestion relief and predict post‐discharge patients' outcomes.^152^

Diuretic resistance and, more generally, persistent congestion despite medical treatment is the main limitation to current treatment of AHF. Diuretic resistance is mainly caused by tubular mechanisms rather than insufficient delivery.^153^ Combination with a thiazide diuretic or metolazone may be effective though with increased rate of worsening renal function and electrolyte abnormalities.^154^ Other diuretic and aquaretic strategies, such as with the combination of high dose spironolactone or with tolvaptan, have not been effective.^155^, ^156^ However, tolvaptan has been associated with favourable outcomes in specific subsets of patients, such as those with diuretic resistance and hyponatraemia, especially in Eastern Asian countries.^11^, ^157^, ^158^ Ultrafiltration and renal replacement therapies are indicated in patients who do not respond to diuretics and with renal failure.^2^ Ultrafiltration has been tested as an alternative to loop diuretics and a new trial is ongoing.^159^ Trials with short‐term administration of new intravenous drugs, such as ularitide or serelaxin, have failed to show an effect on post‐discharge outcomes.^160^, ^161^ However, an effect on in‐hospital clinical course, including events such as in‐hospital worsening HF, might be possible.^49^, ^160^ New intravenous agents with potential favourable effects are under study.^161^, ^162^

## Devices

### Cardiac resynchronization therapy

Prediction of response to cardiac resynchronization therapy (CRT) is still an area of intense research. A meta‐analysis including 1591 patients from three double‐blind, randomized trials demonstrated that only a longer QRS duration and a lower LVEF were independent predictors of a better clinical response to CRT, defined as an improvement in clinical composite score at 6 months.^163^

The lack of response to CRT may be explained by an insufficient amount of cardiac resynchronization due to a large scar and/or difficulties in implantation. The usefulness of multimodality cardiac imaging as a guide to CRT implantation was investigated in two controlled studies in CRT candidates. In the first study, the non‐scarred myocardial segment with the latest mechanical activation was identified by 99m Technetium myocardial perfusion imaging, for vitality, and speckle‐tracking echocardiography, for dyssynchrony. Then, cardiac computed tomography venography was used to select the sinus branch closest to the centre of the optimal pacing site.^164^ The second study used cardiac magnetic resonance imaging to detect non‐scarred myocardial areas and longitudinal myocardial strain imaging by speckle‐tracking echocardiography to identify the area with the greatest dissynchrony. The primary endpoint was, in this case, a ≥15% reduction in LV end‐systolic volume and was reached in 78% of the patients assigned to the imaging modality vs. 56% of those who underwent lead placement with the routine procedure. These approaches yielded better lead positioning and an increased proportion of responders to CRT.^165^

Right ventricular pacing can adversely affect LV function, determining a progressive inter‐ventricular dyssynchrony. A study performed by Burns *et al*.,^166^ which involved patients from the adaptive CRT trial with normal atrioventricular conduction (≤200 ms during sinus rhythm), compared the chronic effects of CRT on LVEF, assessed by echocardiography, when biventricular pacing or LV pacing alone was used. It was found that in patients with normal atrioventricular conduction timing, LV‐only pacing with native RV activation, through the adaptive CRT algorithm, may improve LVEF and global LV radial strain compared with biventricular pacing, because of better apical and septal function.^166^

### Implantable cardioverter defibrillators

Despite guideline recommendations, evidence for a benefit of prophylactic implantable cardioverter defibrillator (ICD) implantation is still debated, namely, in patients with non‐ischaemic cardiomyopathy. The Danish study (Danish Study to Assess the Efficacy of ICDs in Patients With Non‐Ischaemic Systolic Heart Failure on Mortality) failed to demonstrate an overall effect on all‐cause mortality with prophylactic ICD implantation in patients with HFrEF caused by non‐ischaemic disease.^167^ A large meta‐analysis including 40 195 patients from 12 randomized clinical trials showed a 44% decline in the rate of sudden death across the trials, suggesting beneficial effects of evidence‐based therapy also on sudden cardiac death rate.^168^ To date, further investigation better criteria for the selection of patients for ICD implantation, beyond the simple LVEF criterion, seems needed. Further evaluation in prospective clinical investigations is warranted.

## Disease management

Single studies have been often unsuccessful in showing benefits of disease management programmes and insufficient number of patients studied may have had a role. Van Spall *et al*.^169^ performed a systematic review and network meta‐analysis of randomized trials published in 2000–2015 comparing transitional care services with usual care in patients with a recent hospitalization for HF. Their analysis included 53 studies and 12 356 patients. Among all the disease management programmes, nurse home visits were the most effective to reduce post‐discharge all‐cause mortality, compared with usual care [relative risk (RR) 0.78, 95% CI 0.62–0.98], followed by disease management clinics (RR 0.80, 95% CI 0.67–0.97). Nurse home visits were also the most effective to reduce all‐cause readmissions (RR 0.65, 95% CI 0.49–0.86), followed by nurse case management and disease management clinics. Nurse home visits also had the best cost‐efficacy ratio.^169^

Resource utilization may be critically dependent on co‐morbidities and patients' complexity.^170^, ^171^ A psychogenetic determinant of worse clinical outcomes and healthcare utilization was recently assessed in patients with a recent acute decompensation of HFrEF. Neuropeptide S works through the G‐protein‐coupled neuropeptide S receptor pathway and plays a strategic role in the regulation of anxiety, with T‐allele carriers exhibiting a cognitive overinterpretation of fear reaction. Angermann *et al*.^172^ demonstrated higher all‐cause death and re‐hospitalization in homozygous carriers of the gain‐of‐function T‐allele (TT genotype) carriers (*n* = 198), who were prone to develop an exaggerated perception of somatic symptoms and greater anxiety, if compared with AT/AA carriers (*n* = 726).

### Telemonitoring

The MORE‐CARE (MOnitoring Resynchronization dEvices and CARdiac patiEnts) study was an international, prospective, multicentre, randomized controlled trial assessing the efficacy of telemonitoring in 865 patients undergoing CRT defibrillator implantation. Remote monitoring did not reduce mortality or cardiovascular and device‐related hospitalizations, compared with in‐office follow‐ups alone. However, a significant 38% reduction of healthcare resource usage was found, mainly driven by the marked reduction of in‐office visits.^173^

### Adherence to treatment

There is still insufficient awareness of the importance and severity of HF.^174^ Increasing awareness and adherence to treatment is a major component of all disease management programmes. Adherence to evidence‐based treatment, both with respect to drug administration and dosage, was shown to be an independent predictor of outcomes in a large prospective multicentre study in 6669 outpatients with HFrEF. Good adherence to treatment was associated with better outcomes at multivariable analysis. In contrast, poor adherence to treatment, 22% of the studied patients, was associated with higher overall mortality (RR 2.21, 95% CI 1.42–3.44), as well as cardiovascular mortality (RR 2.27, 95% CI 1.36–3.77), HF mortality (RR 2.26, 95% CI 1.21–4.2), and combined cardiovascular or HF hospitalization or death, at 6 month follow‐up.^175^

In BIOSTAT‐CHF, a study where optimization of medical treatment with the initiation of evidence‐based treatment and its uptitration was prospectively indicated, only 22% and 12% of the study patients reached the target dose of angiotensin‐converting enzyme inhibitor/angiotensin receptor blockers and beta‐blockers, respectively. Lack of reaching ≥50% of the recommended dose of these drugs because of symptoms, side effects, and non‐cardiac organ dysfunction was associated with an RR of mortality rate of 1.72 (95% CI 1.43–2.01) for angiotensin‐converting enzyme inhibitor/angiotensin receptor blocker, and of 1.70 (95% CI 1.36–2.05) for beta‐blocker. No difference in the risk of death and/or HF hospitalization was found between patients who reached 50–99% and those reaching ≥100% of the target dose.^51^, ^176^ Undertreatment with MRAs was also noted with only 56% of the patients without contraindication receiving them and 16% discontinuing them during follow‐up.^177^ Variables, such as hypokalaemia and abdominal obesity, which may amplify or enhance, respectively, the favourable effects of MRA have been identified in retrospective analyses of previous trials.^120^, ^178^ In SHIFT, non‐adherence to treatment was independently associated with an increase in the primary composite endpoint of cardiovascular death and HF hospitalization, as well as other outcomes, independently of treatment allocation.^179^ In a nationwide prospective cohort study from Sweden, including 231 437 patients with new‐onset HF, enrolment in an HF registry was associated with a 35% lower mortality (RR 0.65, 95% CI 0.63–0.66). However, this effect was eliminated after adjustment for demographic variables and cardiovascular and HF medical treatment, thus showing the major role of therapy implementation and adherence to therapy for the beneficial effects of patients' registries.^180^

### Palliative care

Palliative care in HF is still largely underdeveloped. Even in countries with a well‐developed healthcare system, only around 4% of patients are referred for specialist palliative care, whereas a large proportion of patients and their families would benefit from receiving specialist palliative care support.^171^ The Palliative Care in Heart Failure (PAL‐HF) study was a randomized, controlled, two‐arm trial aimed to assess the impact of an interdisciplinary palliative care intervention combined with usual HF management on HF‐related and overall quality of life in a population of 150 patients with advanced HF. The palliative care programme adds favourable effects on physical, psychosocial (anxiety/depression), and spiritual quality‐of‐life measurements, compared with usual care alone.^181^

## Mechanical circulatory support

### Temporary support

Venoarterial extracorporeal membrane oxygenation (VA‐ECMO) can improve survival of critically ill patients with cardiogenic shock and severe HF. The applicability of such a mechanical support and its potential benefit on peripheral circulation may be limited by increased LV afterload, secondary to retrograde blood flow. Many modalities of LV unloading may be used to overcome this limitation.^182^ Pappalardo *et al*.^183^ studied 157 patients with cardiogenic shock, 123 treated with VA‐ECMO alone and 34 with concomitant Impella. The two groups were compared by propensity matching. Concomitant treatment with VA‐ECMO and Impella improved outcomes, compared with VA‐ECMO alone, with a lower in‐hospital mortality and a higher rate of successful bridging to either recovery or further therapy, compared with VA‐ECMO alone.^183^

## Permanent left ventricular assist devices

The improvement in the technical characteristics of LV assist devices (LVADs) is broadening their indications to a larger number of patients with advanced HF, either as a bridge‐to‐transplantation or as destination therapy.^2^, ^184^

The new magnetically levitated centrifugal continuous‐flow pump was compared with the axial continuous‐flow pump in a prospective randomized trial in 294 patients with advanced HF. The primary endpoint was a composite of survival free of disabling stroke and of reoperation to replace or remove the device at 6 months after implantation. It occurred in 131 patients (86.2%) in the centrifugal‐flow pump group and in 109 (76.8%) in the axial‐flow pump group with the difference caused by less frequent reoperation for pump malfunction in the centrifugal‐flow pump group than in the axial‐flow pump group (0.7% vs. 7.7% at 6 months). Suspected or confirmed pump thrombosis occurred in no patients in the centrifugal‐flow pump group and in 14 patients (10.1%) in the axial‐flow pump group.^185^ At 2 year follow‐up, the primary endpoint of survival free of disabling stroke or of reoperation occurred in 151 patients (79.5%) in the centrifugal‐flow pump group, as compared with 106 patients (60.2%) in the axial‐flow pump group (RR 0.46, 95% CI 0.31–0.69). Reoperation for pump malfunction was less frequent in the centrifugal‐flow pump group than in the axial‐flow pump group (three patients, 1.6%, vs. 30 patients, 17.0%). Similarly, the rate of stroke was lower in the centrifugal‐flow pump group than in the axial‐flow pump group (10.1% vs. 19.2%). The rates of death and disabling stroke were similar in the two groups.^186^

Heart transplantation or LVADs are still underused in patients with advanced HF. The ScrEEning for advanced HF treatment (SEE‐HF) study was conducted on 1722 patients screened at eight centres in seven European countries. The proportion of patients eligible for heart transplantation or LVAD was low (*n* = 99, 5.7%). However, this indication was unrecognized in 26% of these patients.^187^

The identification of LVAD candidates at risk for RV failure is a major unmet need. A meta‐analysis of 36 studies including 995 of 4428 patients with post‐LVAD implantation RV failure identified as preoperative predictive factors continuous renal replacement therapy, ventilatory support, high NT‐proBNP, international normalized ratio and white blood cell count, high central venous pressure, low RV stroke work index and mean arterial pressure, high RV/LV ratio, and low longitudinal systolic strain of the RV free wall.^188^

## Novel treatments

New drugs for the treatment of HF were considered in the previous chapters. We summarize here non‐medical treatment of potential interest for HF patients.

### Stem cell therapy

Many Phase II studies were recently completed with stem cells. These included muscle‐derived stem cells with connexin‐43 overexpression,^189^ autologous bone marrow‐derived cells with or without granulocyte colony‐stimulating factor administration,^190^ cardiopoietic cells,^191^, ^192^ allogeneic mesenchymal stem cells,^193^ and umbilical cord mesenchymal stem cells.^194^ *In situ* reprogramming of endogenous cardiac fibroblasts, physiologically involved in tissue remodelling, into functional cardiomyocytes appears to be a novel future therapeutic approach.^195^

### New devices

An interatrial shunt device has been used to lower increased left atrial pressure in HF patients. This was tested first in 10 HFrEF patients in a proof‐of‐concept study. Its implantation was associated with a decrease in PAWP with no changes in PAP or right atrial pressure and with improvements in New York Heart Association classification, quality of life, and 6 min walk test distance.^196^ A Phase 1 study in 68 patients with HFpEF showed a reduction in PAWP both at rest and during exercise with an increase in exercise duration.^197^ Similar results were found in a randomized, sham‐controlled, multicentre trial.^198^ One year follow‐up of these studies showed persistent patency of the device, sustained improvement in symptoms, quality of life, and exercise tolerance, and possible favourable effects on hospitalizations and mortality.^199^, ^200^, ^201^

Given the pivotal role of the autonomic nervous system in the progression of HF, specific therapies seem warranted.^202^ Randomized controlled trials with vagal nerve stimulation have given mostly neutral results, to date. However, this might have been caused also by limitations in the modality of vagal nerve stimulation as well as by patient selection.^202^ Baroreceptor stimulation causes both sympathetic inhibition and parasympathetic activation. Baroreceptor response is blunted in patients with HF. Hence, baroreceptor activation through electrical stimulation of the carotid sinus nerve distal to the mechanoreceptor seems a promising treatment to restore sympatho‐vagal balance in patients with HF.^202^ Initial studies have given favourable results. Namely, in a multicentre, randomized, controlled, open‐label trial in 146 patients with HFrEF, patients on baroreceptor activation for 6 months had a greater improvement in New York Heart Association class, quality of life, and 6 min walk test distance and reduced NT‐proBNP levels, though no change in LVEF.^203^ A larger trial, BeAT‐HF (Barostim therapy for HF, Clinicaltrials.gov ID: NCT02627196) is ongoing.^202^

Augmented reflex responses from peripheral chemoreceptors contributes to sympathetic activation and excessive ventilatory response to exercise in HF. Unilateral surgical carotid body resection reduced sympathetic activity and the ventilatory response to exercise and improved exercise capacity in a first‐in‐man study in 10 patients with HFrEF.^204^ Spinal cord stimulation was tested in a prospective, multicentre randomized, parallel, single‐blind, controlled study in 81 HFrEF patients. The treatment failed to reduce LV end‐systolic volume, primary endpoint of the trial, as well as other secondary endpoints.^205^ Phrenic nerve stimulation for the treatment of central sleep apnoea improved sleep metrics and quality of life with a numerical reduction in hospitalizations in patients with HF.^206^, ^207^

## Concluding remarks

Last year has witnessed major advances in all the aspects of the diagnosis, follow‐up, and treatment of patients with HF. It is difficult to say if there is an area whose results are more promising. New drugs are unlikely to be effective for all patients with HF. Major heterogeneity is emerging, above all for patients with HFpEF and AHF and also for those with HFrEF. Intensive research, including the use of more advanced proteomic and genomic techniques, is likely to allow better characterization of single patients. However, looking more closely, we are left with different clinical presentations and co‐morbidities. Co‐morbidities are easy to detect and have a clear impact on the HF patients' clinical course. To date, their detection and proper treatment seem among the best options to deliver personalized treatment to HF patients. In some cases, such as diabetes and iron deficiency and also in the case of cardiovascular co‐morbidities such as atrial fibrillation or valve disease, it is possible that the largest progress for HF prevention and treatment will come from therapies targeted at these specific co‐morbidities rather than treatments applicable to all the HF patients.

## Acknowledgment

We wish to thank Paola Luciolli for help with proof correction and manuscript language editing.

## Conflict of interest

M.S.A. reports receiving personal fees from Servier. M.M. has received consulting honoraria from Bayer, Fresenius, and Novartis.
